# The New Generation from Biomembrane with Green Technologies for Wastewater Treatment

**DOI:** 10.3390/polym10101174

**Published:** 2018-10-22

**Authors:** Ahmed Mohamed El-hadi, Hatem Rashad Alamri

**Affiliations:** 1 Department of Physics, Faculty of Applied Science, Umm Al-Qura University, Al-Abidiyya, P.O. Box 13174, Makkah 21955, Saudi Arabia; 2 Department of Basic Science, Higher Institute of Engineering and Technology, El Arish, North Sinai 9004, Egypt; 3 Physics Department, Jamoum University College, Umm Al-Qura University, Makkah 21955, Saudi Arabia; PHBhadi1963@yahoo.com

**Keywords:** PLLA nanofibers membranes, electrospinning, PPC, PHB, TEC as plasticizer, biopolymer blends, water purification

## Abstract

A biopolymer of polylactic acid (PLLA)/polypropylene carbonate (PPC)/poly (3-hydroxybutrate) (PHB)/triethyl citrate (TEC) blends was prepared by the solution-casting method at different proportions. The thermal characteristics were studied by differential scanning calorimetry (DSC) and thermogravimetry (TG). PHB and TEC were added to improve the interfacial adhesion, crystallization behavior, and mechanical properties of the immiscible blend from PLLA and PPC (20%). The addition of more than 20% of PPC as an amorphous part hindered the crystallization of PLLA. PPC, PHB, and TEC also interacted with the PLLA matrix, which reduced the glass transition temperature (*T*_g_), the cold crystallization temperature (*T*_cc_), and the melting point (*T*_m_) to about 53, 57 and 15 °C, respectively*.* The *T*_g_ shifted from 60 to 7 °C; therefore, the elongation at break improved from 6% (pure PLLA) to 285% (PLLA blends). In this article, biomembranes of PLLA with additives were developed and made by an electrospinning process. The new generation from biopolymer membranes can be used to absorb suspended pollutants in the water, which helps in the purification of drinking water in the household.

## 1. Introduction

Important research has been undertaken to mix polylactic acid (PLLA) with various natural fibers such as silk, and sisal fibers [1], biopolymers such as poly(vinyl alcohol) (PVA) [2], poly (caprolactone) (PCL) [3], poly (butylene carbonate) (PBC) [4], as well as fillers such as titanium dioxide [5], calcium carbonate [6], and zinc oxide [7]. However, the mechanical properties of PLLA have not improved. Some interesting results in miscible PLLA blends and others were immiscible with poor mechanical properties. It has been added as a compatibilizer such as maleic anhydride to improve the miscibility of PLLA blends, and therefore improve the mechanical properties. However, all of the attempts found that the elongation at break of PLLA blends was very low with limited applications in industry. Therefore, it is necessary to find a method to improve the mechanical properties of PLLA that can be more economical and effective in fabrication, which was the general purpose of this study. There is now a rising demand for biopolymers, which are manufactured from renewable source materials, biodegradable, and do not cause any problems for the environment [8]. PLLA has been greatly considered, as it is biodegradable as well as non-toxic to the environment and the human body [9]. PLLA is brittle and has very low elasticity, which limits its application as a general purpose plastic. It is known that the brittleness of PLLA is due to its slow crystallization rate, which itself is due to the formation of a large spherulite with cracks [10] and a higher a glass transition (*T*_g_ is equal 60 °C), limiting its practical applications in industry. Therefore, many efforts have been made to improve the crystallization process of PLLA by adding PHB as biomacromolecules [8,11]. Physical polymer blends [12,13,14,15,16] are one of the most economical methods of developing new polymers with improved physical properties for industrial applications. As the miscibility of polymer blends is one of the major effects on the properties of such blends, focus on the miscibility of polymer blends has increased [17]. It is one of the most effective and promising methods to increase the crystallization rate for PLLA, alongside adding plasticizers [8,18] to reduce the *T*_g_. PLLA has been selected for biomedical applications because of its full biodegradability ability, and its degradation products are non-toxic and biocompatible. Polypropylene carbonate (PPC) is a rubbery amorphous thermoplastic produced by the copolymerization of propylene oxide and carbon dioxide. PPC is biodegradable and has excellent mechanical properties such as high flexibility, high elongation at break, and toughness [19]. PPC is used as binder resins and packing materials; however, PPC has poor thermal stability. A combination of PLLA, PPC, PHB, and a plasticizer at different ratios as a biopolymer blend has been used to obtain suitable properties for use in tissue engineering, food packaging applications [11], and the filtration process.

The aim of this study was to manufacture a membrane for cleaning wastewater, especially in the field of filtering technology in households, with a very thin diameter [20,21]. The mechanical properties of these PLLA blends are the important criteria to determine their applications in industry, specializing in biofilters for water treatment. The mechanical behavior of pure PLLA and its blend was calculated to examine the effect of additives in the PLLA blend. Plasticizers act similar to diluents when mixed with the polymer. Plasticizers have a low molecular weight, which leads to an increase in the free volume between molecule chains. As a result, the glass transition temperature is lower, increases the elongation at break, and reduces the tensile strength [22,23,24].

Water is one of the most natural important human resources due to the large population growth in developing countries, which has caused changes in the lifestyle. The request for drinking water and for all of the purposes in the house as well as harvesting large amounts of wastewater from the home or industrial unit has increased over time. This has caused big problems in the pollution of water resources when it is cast into rivers, seas, or in groundwater, causing serious health problems in most developing countries for humans and animals. One of the most important challenges raised by population growth is that the government must provide fresh water for all of these people. Consequently, there is a need for innovative new technology to improve the treatment of drinking water purification and sewage treatment. A membrane technology made of nanofibers can help in improving the efficiency of eliminating contaminations in the water [25,26,27,28,29].

One of the most important methods of producing nanofibers of various sizes and shapes of biopolymers is electrospinning, because it produces fibers with different diameters and a large surface area to volume ratio, which makes it capable of industrial applications such as solar cells, fuel cells, and biosensors, as well as medical purposes such as drug delivery, tissue engineering, and surgical suture. Electrospinning [20,21] is an easy, fast, and cost-effective process for producing fibers from 3 μm to 500 nm in length. In the electrospinning process, a drop of the polymer solution is placed at the end of a needle by a syringe pump with a small flow rate. Voltage is then applied to the polymer solution, producing electrical charges on the tip of the needle to overcome the surface tension, which are pulled by the high voltage. Then, at the end, they are collected on the ground collector to the manufactured membrane. Through this process, the size of the pores of the membrane is very small, less than 1 nm, to output particles larger than the water molecules.

The aim of the study was to investigate the effect of the additives poly (3-hydroxybutrate) (PHB), PPC, and plasticizer with different weight percentages on the toughness, flexibility, and biodegradation of PLLA. The main purpose of this project was to manufacture PLLA nanofiber membranes for the purification of water and the removal of all of the contaminants from wastewater by a pump to flow water in the membrane, so the mechanical properties of the nanofiber membrane must be good. The blends were characterized by differential scanning calorimetry (DSC), thermogravimetric analysis (TGA), scanning electron microscopy (SEM), and tensile testing. The PLLA films were mechanically tested to obtain their elongation at break and stress strength. The novelty of this project is firstly the improvement of the mechanical properties and the crystallization process of PLLA by addition of PPC, PHB, and triethyl citrate (TEC), which is evident by increasing the elongation at break and reducing *T*_g_, *T*_cc_ and *T*_m_, and then manufacturing the nanofiber as biomembrane. This biomembrane makes better filters for applications in purification water than the traditional petrochemical membrane. This membrane is considered as most promising for purification, because it decomposes after more than two months.

## 2. Experimental

### 2.1. Materials and Blend Preparation

PLLA with a molecular weight *M_w_* = 2.23 × 10^5^ g/mol and PPC with average *M_w_* = 0.5 × 10^5^ PHB, with *M_w_* = 2.33 × 10^5^ g/mol, by Gel permeation chromatography (GPC)and TEC were provided by Sigma-Aldrich Chemicals Ltd. (St. Louis, MO, USA).

The solutions for the casting blends were prepared by dissolving all of the components in dichloromethane (DCM), and then pouring them into a petri dish. After the solvent evaporated, the obtained films were dried in an oven at 60 °C for 24 h, i.e., PLLA + PPC (20%) with a plasticizer (15%–20%) + PHB (10%) were dissolved in dichloromethane (DCM) and left to dry to form a cast film, and then dissolved again in dichloromethane with a constant concentration (24%) of blend 3 to manufacture electrospun fibers. The samples of the PLLA/PPC/PHB/TEC blends were prepared with different weight ratios, as shown in Table 1. The solution casting film of the PLLA blends was manufactured for biodegradation testing (blend 3) and mechanical testing (all of the samples) by melting in a hot hydraulic press, i.e., the cast evaporated film was cut into small pieces and melted between two sheets of aluminum foil in the hot hydraulic press between 170 and 180 °C without pressure, and one minute at 20 KN. The samples were cooled between metal plates.

The blends that were prepared are shown in Table 1.

The samples of blend 3 were manufactured only and then tested for electrospun fibers. The fibers are collected on the fixed aluminum foil with the following parameters such as needle diameter, applied voltages, DCM, flow rate, and the distance between needle and target: 1.3 mm, 25 kV, 24 wt %, 0.25 μm·h^−1^, and 15 cm, respectively.

### 2.2. Methods

#### 2.2.1. Differential Scanning Calorimetry (DSC)

Thermal analysis was carried out from −50 to 200 °C at the heating and cooling rates of 10 °C· min^−1^ using a differential scanning calorimeter (Shimadzu-DSC 50, Kyoto, Japan). Samples with a weight of 400 ± 100 μg were sealed in aluminum sample pans and kept under a dry nitrogen atmosphere. The analysis of the DSC curves was carried out for the second heating run data to determine the glass transition temperature (*T*_g_), the melting temperature (*T*_m_), and the cold crystallization temperature (*T*_cc_).

#### 2.2.2. Mechanical Analysis

The mechanical properties of the PLLA blend films were tested using a Shimadzu universal testing machine equipped with a 10-kN load cell and interfaced with a computer. All of the samples were cut in a dumbbell shape, similar to dog bone specimens, (Dumb Bell Ltd. SDL-100, Kawagoe Japan) with dimensions in length of 80 mm, width of 6 mm, and a thickness of 0.1 mm. Tensile tests were performed at room temperature at a crosshead speed of 5 mm·min^−1^ (in agreement with ASTM D882). Five specimens of each formulation were tested, and the average values were described. From the relation between stress σ (in Pa) and elongation ε (in %), ε = (*L*_0_ − *L*)/*L*, *L*_0_ = original length, *L* = length after elongation. Finally, the fracture surface of the electrospun samples was investigated by SEM.

#### 2.2.3. Thermogravimetric Analysis (TGA)

Thermogravimetric analysis for the pure PLLA and its blends were tested using a TGA (Q500) instrument. Sample weights between 10–20 mg were heated under a nitrogen flow (90 mL/min) from room temperature to 600 °C with the heating rate of 40 °C/min.

#### 2.2.4. Biodegradation Test

To study the biodegradation in this paper, we only used a blend 3 film, and not a membrane. The film with dimensions of 2 cm × 2 cm × 0.1 cm was placed inside a small empty bottle of Polyethylene terephthalate (PET) with holes to enter the bacteria, and placed in the wastewater tank in the house for more than two months. Then, the film was removed from the flask and washed with distilled water, dried in an oven at 100 °C, and examined using SEM.

#### 2.2.5. Electrospinning Equipment

PLLA blends were dissolved in different chemical solvents with different concentrations and located in a plastic syringe (10 mL) attached to a needle with an inner diameter (ID) of 1.3 mm. Electrospinning was conducted at room temperature with a high-voltage power supply from the USA (model No. ES60P-20W, Gamma High Voltage Research, Orlando, FL, USA). All of the fibers were pure PLLA, and the blends that were collected on the aluminum foil. A syringe pump (No. BS-9000-USA, Braintree Scientific, Braintree, MA, USA) was used to feed the polymer solutions into the needle tip. The electrospun fibers were collected on a grounded collecting plate. When the electrical voltage increased, the fiber diameter was reduced due to the increased elongation of the jets by the electric field, which also causes an increase in the flow mass of the polymer.

#### 2.2.6. Scanning Electron Microscopy (SEM)

The surface morphology of the PLLA blends nanofibers was observed using a scanning electron microscope (JSM-6360LA, JEOL Co., Boston, MA, USA) at an accelerating voltage of 3 kV. The samples were sputter-coated with gold for 120 s to a thickness of 2–3 nm using a sputter coater (EMITECH K550X, Kent, UK). Images of several sample fibers were obtained using SEM to measure the fiber diameter. The samples were coated with gold.

## 3. Results and Discussion

### 3.1. Differential Scanning Calorimetry (DSC) Analysis

**Figure 1:**
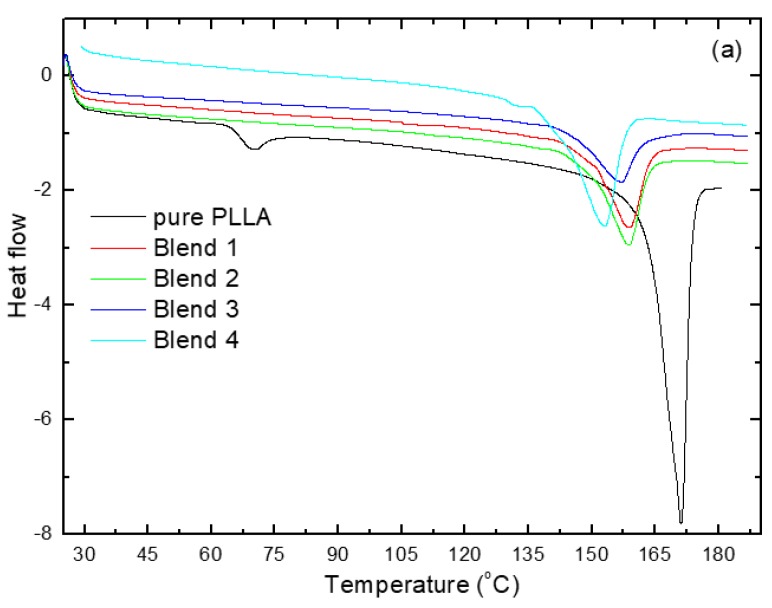

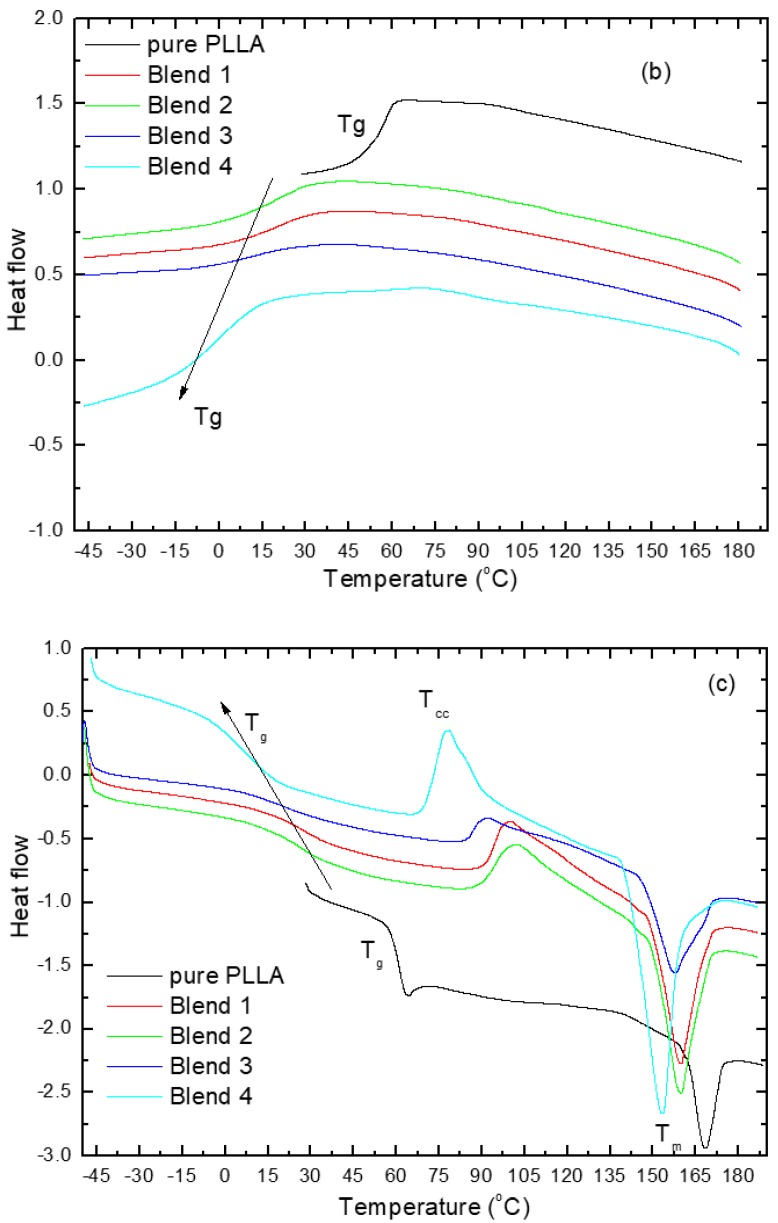
Differential scanning calorimetry (DSC) of pure PLLA and its blends 1,2, 3 and 4: (**a**) the first heating run; (**b**) the cooling from melting; and (**c**) the second heating run.

The miscibility of the PLLA/PPC/PHB/TEC blends was measured by DSC from the first run, the cooling run from melting, and the second run from cooling at a heating rate of 10 °C·min^−1^. DSC curves were verified for pure PLLA; their blends are shown in Figure 1. Pure PLLA demonstrates a glass transition temperature *T*_g_ at around 61 °C [24] and a melting peak (*T*_m_) at 188 °C. Pure PPC shows a *T*_g_ at around 30 °C [19]. At the first heating, the *T*_g_ was obviously shown for pure PLLA, which indicated that it was amorphous (low crystallinity), but the *T*_g_ of the blends appeared unclear due to the high crystallite form by the addition of PHB to the PLLA matrix. Through the first heating, the rearrangement of the macromolecules became crystallized; therefore, a sharp melting peak was presented. It is known that PLLA is semicrystalline, and crystallizes during heating above 120 °C after melt quenching by cold crystallization, i.e., the chain of PLLA can be moved and reordered during heating by the second heating. Below 60 °C, PLLA is amorphous, rigid, and brittle. PHB is semicrystalline, and is chosen as a nucleating agent to help the crystallization of PLLA, i.e., the crystallization process can take place earlier. The manufacturing of PLLA is improved without thermal degradation, i.e., decreasing the viscosity and increasing the free volume. TEC can exist between the polymer chains. This allows for segmental mobility with less energy. PLLA crystallization was analyzed with and without additives from the first DSC heating test in Figure 1. The *T*_g_ was observed in pure PLLA, but was not shown in the PLLA blends, as the *T*_g_ depends on the thermal history during the manufacture of the sample. The relaxation enthalpy of pure PLLA was observed at the *T*_g_, this has been associated with the physical aging of PLLA. This thermal behavior of the films indicated that a large part of PLLA is amorphous after molding in a hydraulic hot press by a fast cooling rate*.* It can be observed that the *T*_g_ decreased with increased plasticizer content for blend 1 at 27 °C, blend 2 at 27 °C, blend 3 at 21 °C, and blend 4 at 7 °C. It is known that the *T*_g_ of polymer blends is the standard for determining the miscibility. The cold crystallization temperature occurs when the temperature rises to above 120 °C (pure PLLA) and 70 °C (PLLA blends). This is due to the addition of PHB; therefore, the crystallization of PLLA is improved. The cold crystallization temperature (*T*_cc_) was found at 100 °C for blend 1, and shifted to 102 °C for blend 2; it was found at 92 °C for blend 3, and 78 °C for blend 4. The melting temperatures (*T*_m_) of the blends were calculated to be 160 °C for blend 1, 160 °C for blend 2, 158 °C for blend 3, and 153 °C for blend 4, respectively. The addition of PHB and TEC had an influence on the position of the *T*_g_, *T*_cc_, and *T*_m_. The decrease in the *T*_g_, *T*_cc_, and *T*_m_ led to the increase of chain mobility; therefore, the crystallinity was enhanced, as shown in Table 2. This is dependent on both the PHB and plasticizer at different ratios. Pure PLLA and its blends showed no crystallization during the quenching process (cooling run with 20 °C·min^−1^) from melting; consequently, they were completely amorphous due to rapid cooling, showing only the *T*_g_. The cold crystallization temperature *T*_cc_ appeared only at the second heating. PLLA and PPC with plasticizer exhibited a single *T*_g_ behavior during a cooling run and second run. A shift was observed in the *T*_g_, *T*_cc_ and *T*_m_, meaning that there was some physical cross-linking between PLLA, PHB, and TEB.

The crystallinity (X%) of PLLA and its blends was evaluated using Equation (1):

X = ΔH × 100/(ΔH_m_^0^(1 − %wt filler/100)) (1)

where ΔH_cc_ is the enthalpy of cold crystallization for the sample or ΔH = ΔH_m_ − ΔH_cc_ (for second heating curves, as ΔH_m_ is the enthalpy of melting of the sample); ΔH_m_^0^ is the enthalpy of melting at 100% crystalline polymer matrix (93.0 J/g for PLLA [30]; and %wt filler is the total weight percentage of PHB and TEC. After extracting the weight percentage of PHB and TEC, these values corresponded to the crystallinity of the blends. From Table 2, The *T*_g_ of PLLA was reduced after the addition of plasticizers, i.e., the greater the concentration of plasticizer, the greater the decrease in *T*_g_, also TEC decreased the interchain forces in the PLLA matrix, and increased its free volume. The higher concentration of TEC means a higher shift in the crystallization temperature. PPC, PHB, and TEC also interacted with the PLLA matrix, which reduced the *T*_g_, *T*_cc_, and *T*_m_ about 53, 57 and 15 °C, respectively. It can be concluded that pure PLLA, pure PPC, and pure PHB had a *T*_g_ at 60, 21 and 0 °C, respectively. If the plasticizer content (TEC) was 30% by weight and the *T*_g_ reached 7 °C, i.e., about 53 °C, it was below pure PLLA. This is due to the strong interaction between all of the components. As the TEC concentration increased, the *T*_g_ shifted lower, and the transition areas enlarged, i.e., one could see the changes in specific heat capacity. The decreasing of the *T*_g_ in the PLLA blends leads to improvements in the mechanical properties. This indicates that the additives are an active method for increasing the elongation at break of PLLA.

Where the first heating (F.H.), second heating (S.H.), ΔH_m_, ΔH_cc_, and X_(%)_ are the enthalpy of melting, enthalpy of cold crystallization, and weight fraction of PLLA, respectively.

### 3.2. Mechanical Properties

**Figure 2:**
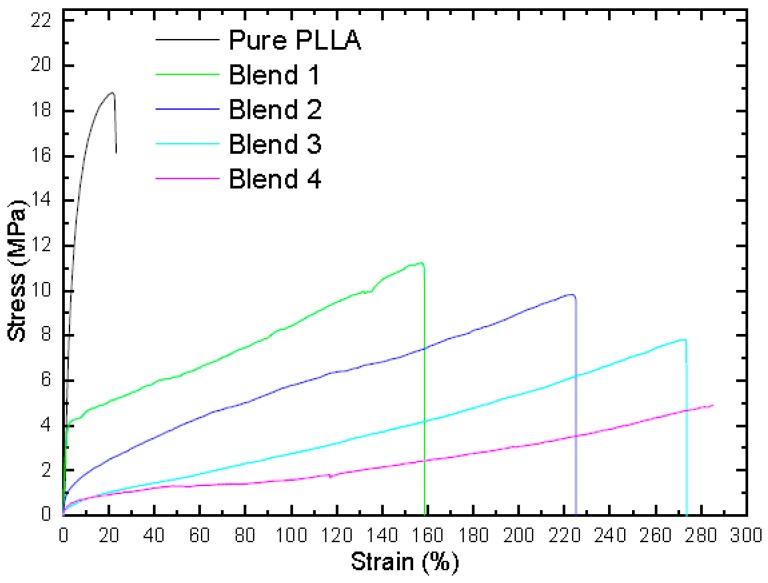
Mechanical testing of stress–strain for PLLA and its blends films at room temperature with a speed of 5 mm/min.

The mechanical properties of the PLLA blends were measured by the study of stress versus strain. Pure PLLA showed a brittle fracture and very low elongation at break 6% and a high tensile strength of 19 MPa, which were in agreement with the results by El-hadi and Silverajah in [24,31]. The stress–strain curves for the PLLA blends are shown in Figure 2, and the tensile strength (σ) and elongation at break (ε) are plotted in Figure 2. It can be seen that as the weight percentage of additives in the blends increased, the tensile strength decreased, i.e., the tensile strength decreased from 11 MPa for blend 1 to 5 MPa for blend 4. The effect of additive content on the fraction of elongation at break for the PLLA blend is shown in Figure 2. The elongation of the PLLA blends was improved with the addition of PPC, TEC, and PHB. The elongation at break in the PLLA blends increased from 158% for blend 1 to 282% for blend 4. The tensile stress decreased from 19 to 5 MPa by increasing the plasticizer with a ratio from 15% to 30%. The decrease in the tensile stress of the blends is due to the low stress of PPC. The combination of PPC with other additives in the PLLA blend improved the strain at break when compared to pure PLLA. It was observed that the elongation at break of the blend-4 film was higher than those of blends 1, 2, and 3. Compared to blend 4, the decrease in elongation at break of blend 1 was related to the decrease in the *T*_g_, besides the shape of the particle size of PPC. It was also found that the *T*_g_ of the PLLA decreased with an increase in the TEC percentage. A small amount of PPC (20%) was added to improve the mechanical properties, and a larger quantity led to an immiscible blend [4]; i.e., when the content of PPC was more than 30%, the blends became immiscible.

The tensile test results indicated a significant improvement in the elasticity and ductility of the PLLA blends, which were well associated with the decrease in *T*_g_ observed in the DSC curves. The enhancement in the mechanical properties of the PLLA blend film was attributed to the presence of PPC in the blend. The mechanical property showed that the entanglement of PPC chains could improve the mechanical properties of the film blend and electrospun fiber membranes by reducing the brittleness of pure PLLA. It can be concluded that there is a two-phase between the PPC and PLLA blends [4]; therefore, the interfacial adhesion between them is very poor, i.e., two glass transition temperatures and poor mechanical properties. The addition of PHB and TEC can improve the interfacial adhesion between PLLA and PPC (20%); therefore, the elongation at break was strongly enhanced as the interfacial adhesion between the two phases was improved.

**Figure 3:**
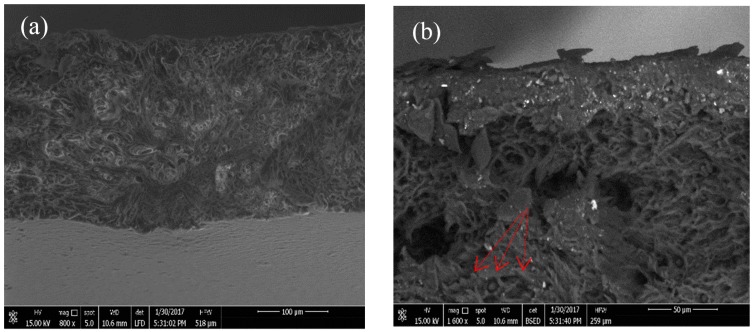

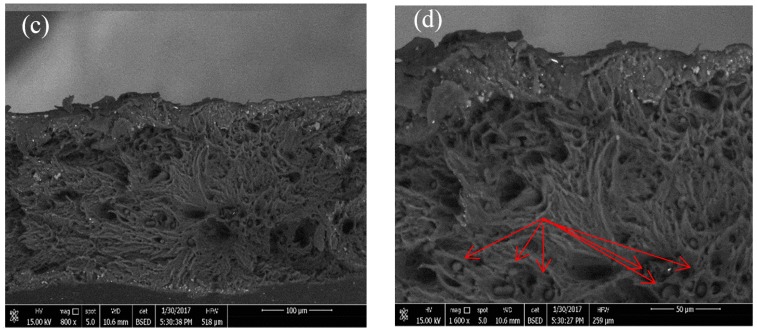
**Figure3.** SEM images of the fracture surface of the blend 1 and 3 films after tensile testing; (**a**,**b**) blend 1; (**c**,**d**) blend 3.

The fracture surfaces of the PLLA blends are shown in the SEM micrographs in Figure 3. It was observed that the fracture surface of the PLLA blends had plastic deformation in the stress direction. It has been shown that PLLA has a brittle fracture surface during the mechanical test [17]. However, the PLLA blends showed different behaviors under tensile testing. The PPC particles act as stress factors on the elastic property in the PLLA matrix. Many particles with holes were observed on the fractured surface, and these voids were magnified along the stress direction, as shown in Figure 3. The fracture surface after the cold drawing process was rough and smooth, where many particles were observed in the holes and were clear with large plastic deformation. During the investigation of the PLLA samples of blends 1 and 3 after tensile testing, the particle size of PPC appeared in the PLLA matrix within approximately 5 µm. This is clearly shown by the red arrows in Figure 3b,d. To conclude, the compatibility between the dispersed PPC phase in the PLLA matrix in the blending process is necessary for toughness. The SEM images of the fractures in blends 1 and 3 can be seen in Figure 3. It was found that the domain size of PPC was between 3–5 µm, which means that PLLA and PPC were partially miscible. No larger domains were found in the PLLA matrix. The addition of PPC, PHB, and TEC had major changes in the elongation at break of PLLA.

### 3.3. Thermogravimetric Analysis (TGA)

**Figure 4:**
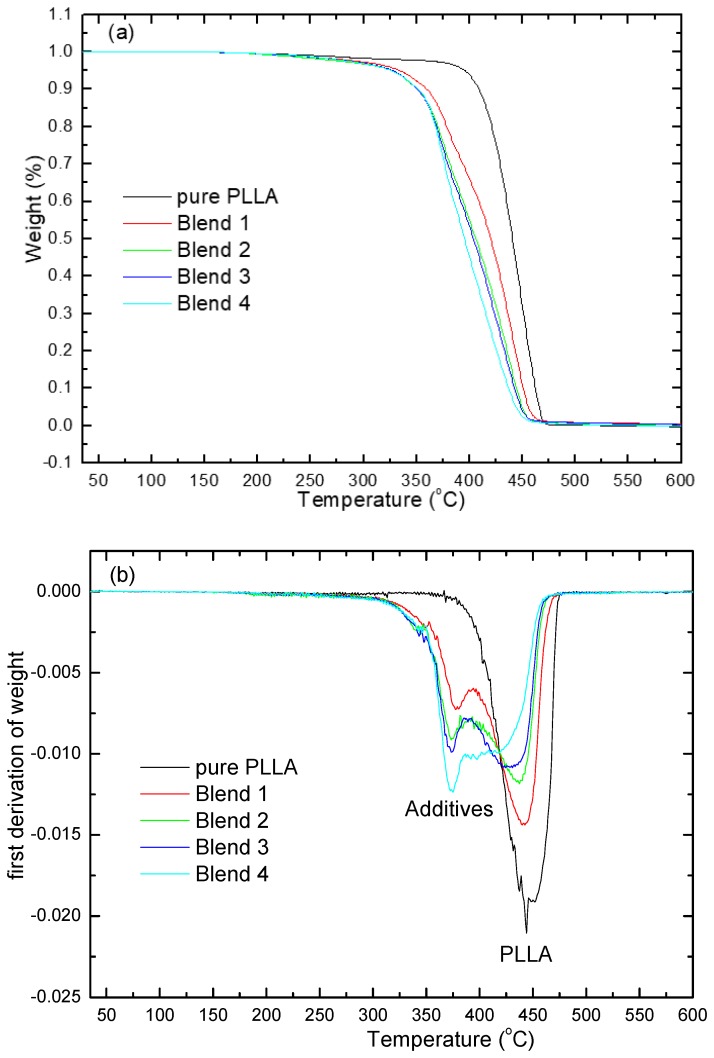
Thermogravimetric analysis of pure PLLA and its blends: (**a**) thermogravimetric curves and (**b**) derivative thermogravimetric curves.

**Figure 5:**
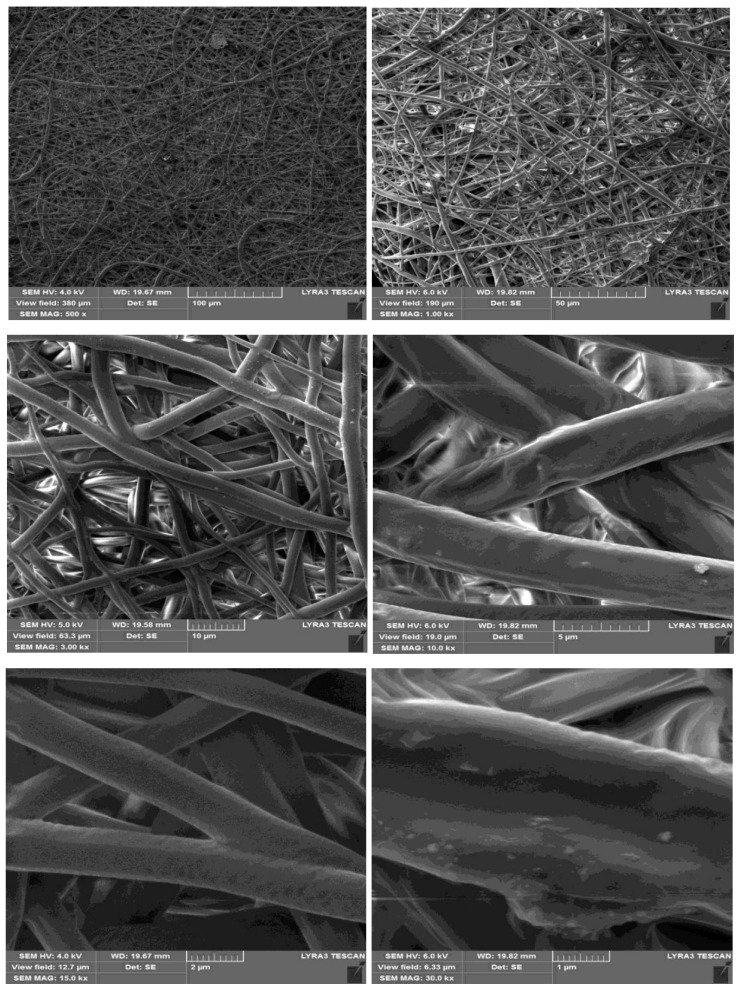
SEM micrographs of the blend 3 electrospun mats at different magnifications.

TGA plays a very important role in determining the thermal stability of polymeric materials. It is a process in which material is decomposed by heating, which causes the breaking of the bonds between the molecules. The thermal stability of PLLA and PPC, and their blends with additives, were investigated using thermogravimetric analysis (TGA). Pure PLLA was stable without major weight loss up to 450 °C, while the blends of PLLA showed a continuous decrease in weight loss at 370 °C. The thermal properties of pure PLLA and its blends were investigated by TGA. The TGA thermogravimetric curves provide information about the degradation of the polymeric materials. Figure 4a,b show the TGA and DTGA results of pure PLLA and its blends with PPC, PHB, and TEC, respectively. From the TGA curves in Figure 4a, there are two major stages in the breakdown of the chain in the PLLA blends. In the region of 370 °C, the first weight loss corresponded to the breakdown of the additives (PPC, PHB, and TEC). The second weight loss in the region of 450 °C corresponded to the decomposition of PLLA. As shown in Figure 5b, pure PLLA had one decomposition peak at approximately 450 °C. After adding PPC, PHB, and TEC, the decomposition temperature of the blends showed a slight change when compared to pure PLLA, but its peak intensity increased with the increasing plasticizer content. The addition of PPC, PHB, and TEC led to a small decrease in the thermal stability of the PLLA blends. This degradation peak at 370 °C corresponded to the degradation of the PPC, PHB, and TEC, which have a lower thermal stability than pure PLLA. Different changes were observed in the PLLA blends with additives in the TGA measurements. This was evident after the addition of the plasticizer TEC in different weights of 15 wt %, 20 wt %, 25%, and 30%. It can be concluded that the thermal degradation of PPC, PHB, and PLLA occurred at 270, 350 and 450 °C, respectively. The thermal decomposition temperature of PLLA with additives decreased from 450 °C (pure PLLA) to 370 °C for the additives PPC, PHB, and TEC with a heating rate of 40 °C·min^−1^.

### 3.4. Fiber Morphology by Scanning Electron Microscopy (SEM)

Electrospinning is obtained when a great voltage is applied to a polymer with solvent. A polymer solution is placed in a syringe that is connected to the positive electrode, and the syringe pump is moved to push the solution. The collector is connected with the negative pole of the ground. The application of the large electric field pulls the solution from the needle to a very small jet and is elongated; then, the solvent is evaporated, which leads to the formation of nanofibers. Figure 5 shows the morphologies of the blend 3 electrospun fibers. The obtained fibers had uniform, smooth, and cylindrical morphology with a diameter between 1–2 μm, and 500 nm without beads.

### 3.5. Biodegradability Test in Wastewater

**Figure 6:**
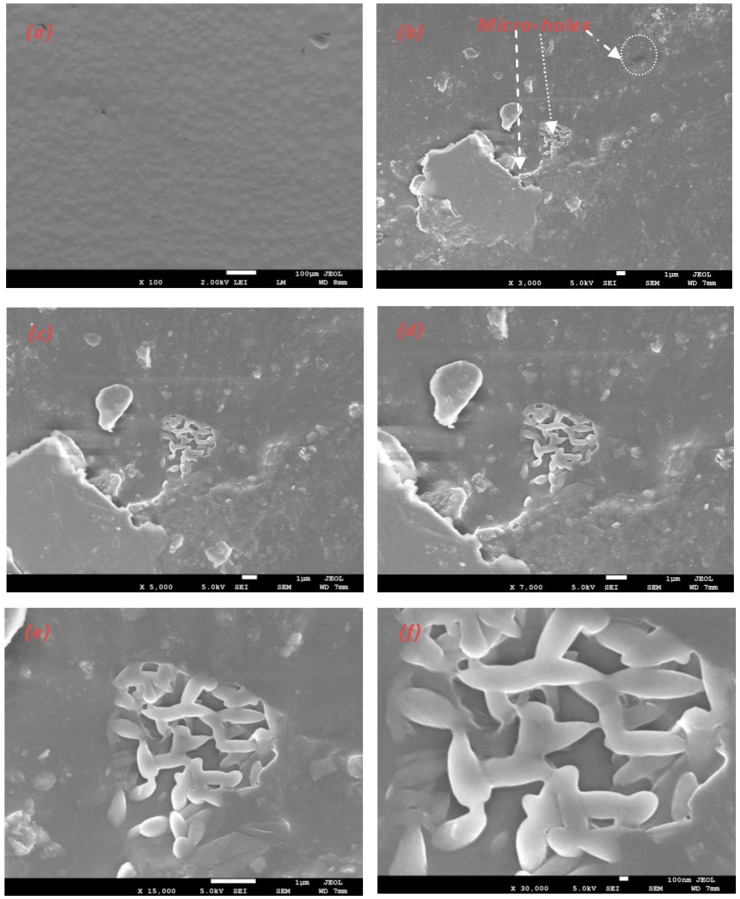
Scanning electron micrograph of the surface images of the blend-3 film; (**a**) before the degradation; (**b**–**f**) after immersion in wastewater for eight weeks with different scales.

There are many methods to measure the biodegradation test on the surface of bioplastics, i.e., the film is placed in the soil, sea water, oceans, or sewage, and compost; and then the film surface is observed by SEM. All of these methods are simple and direct, but there is another method, such as the aerobic test. In this paper the changes in the blend-3 film surfaces were analyzed by SEM before and after biodegradation. It is known that plastic materials can be decomposed through various mechanisms, including mechanical, thermal, UV-photo, and biological degradation. During the degradation of polymers, the physical properties and the chemical properties are changed due to environmental factors such as light, heat, humidity, and biological activity. SEM observations of the blend-3 film before the degradation showed a smooth surface and flat surface (Figure 6a). Bioplastics degradation is caused by bacteria or fungus, where bacteria eat bioplastics as a source of carbon; then, an area appears to grow inside. After being immersed in wastewater for two months, the surface of the sample was found to be degraded in some areas. The surfaces were observed with pores of different sizes in microns. The degradation at the surface began after two months in wastewater. Degraded surfaces were observed, and some pores with different sizes were found. There were some micro holes from the elimination of the amorphous regions from blend 3 rather than the crystalline regions. It is known that most household filters clean the drinking water from petrochemical plastics. This filter is changed every two months, and is put in the waste. As a result, it does not biodegrade, and causes environmental problems. Therefore, a biofilter from a PLLA blend has been manufactured to replace these petrochemical filters. This is the main purpose of this project.

### 3.6. Filtration Test

**Figure 7:**
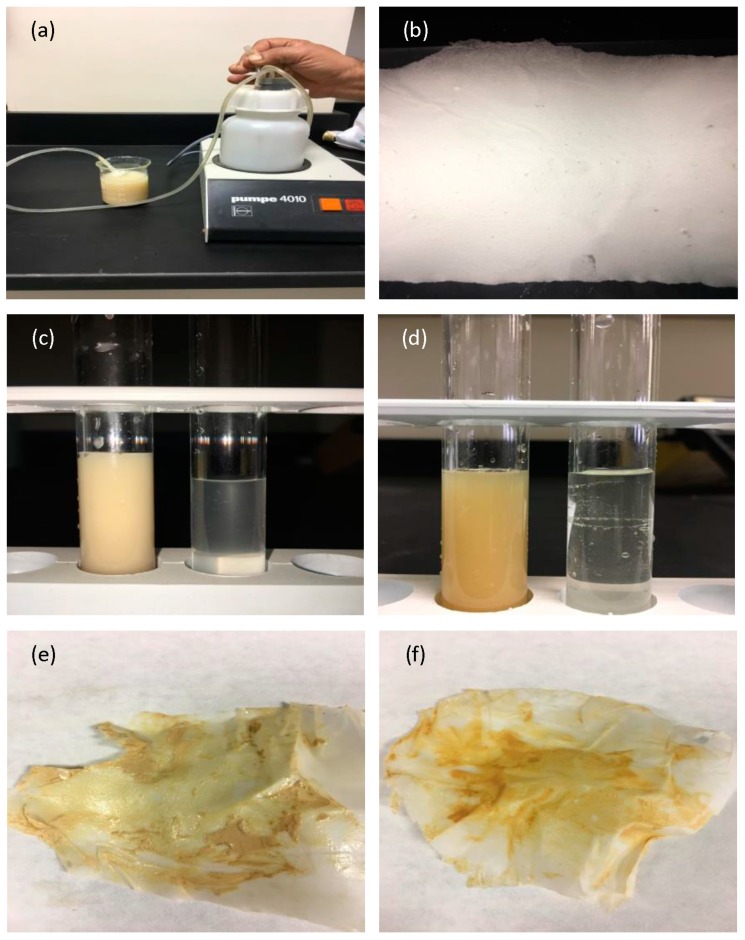
Wastewater treatment processes. (**a**) Pump with unprocessed wastewater; (**b**) The surface of the membrane before the filtration process; (**c**) Direct filtration by the nanofiber membrane from wastewater with very small clays in the Saudi soil; (**d**) Direct filtration by the nanofiber membrane from wastewater with sand from the Saudi Earth; (**e**) Particles at the surface of the membrane after the filtration process from clays; and (**f**) Particles at the surface of the membrane after the filtration process from very small sand.

**Figure 8:**
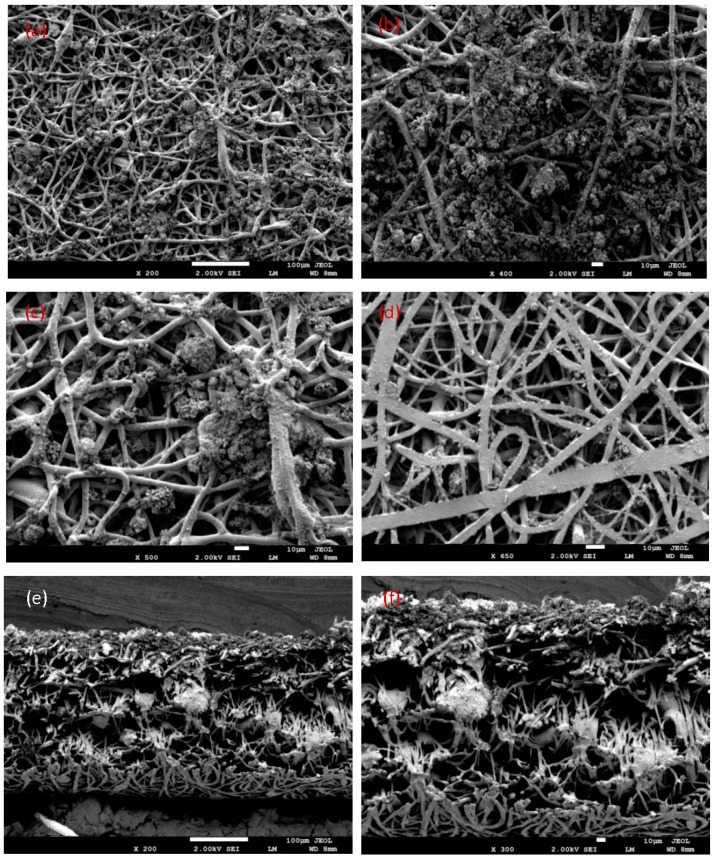
SEM images of nanoparticles and microparticles of clays from the Saudi soil at the surface of the membrane after the filtration process; (**a**–**c**) Top of the membrane layer with cylindrical fibers; (**d**) The bottom membrane layer with flat fibers; (**e**,**f**) Cross-section of the membrane with different scales.

**Figure 9:**
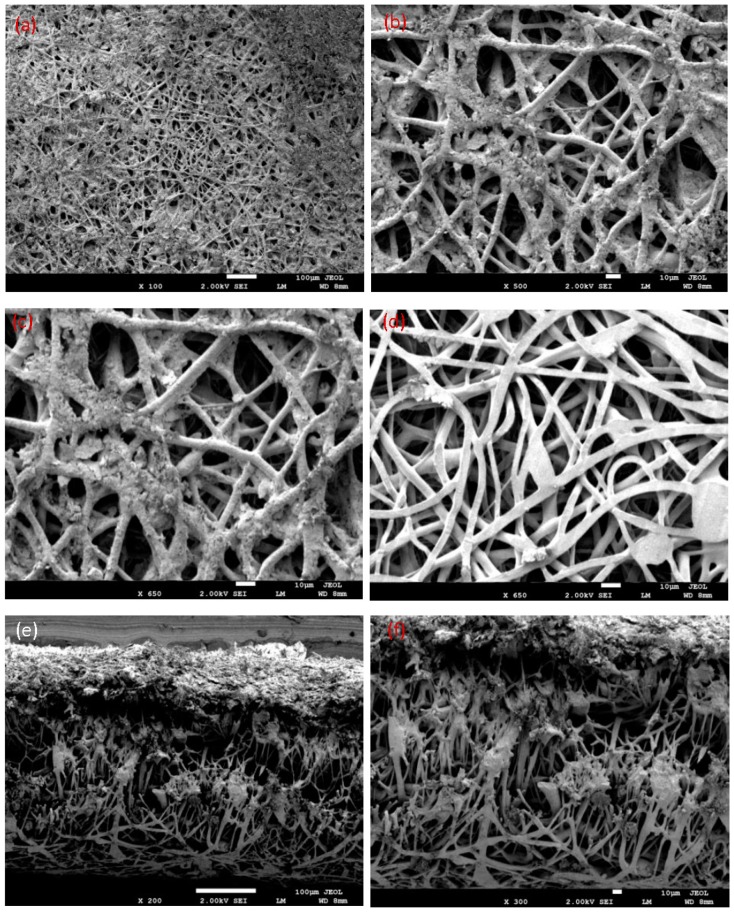
SEM images of nanoparticles and microparticles of fine sand from the Saudi Earth at the surface of the membrane after filtration process; (**a**–**c**) Top membrane layer with cylindrical fibers; (**d**) Bottom membrane layer with flat fibers; (**e**,**f**) Cross-section of the membrane with different scales.

One of the advantages of a membrane mat from nanofibers is the ability to filter the soil, dirt, clays, particles, and microbes from wastewater. The fiber diameter, pore diameter, and filtration efficiency were measured. It is found that the membrane is stable, and its elasticity is higher. The membrane is unbroken, and did not break upon pulling out the wastewater by using pump 4010 with a flow rate of 4 mL/s and speed of 0.566 m/s. This is obvious in Figure 7 before and after the filtering process. The membrane was twisted without any break, whose pores are smaller than the particles to be removed. This is evident when making a cross-section in the membrane and viewing it by SEM in Figure 8 and Figure 9e,f. This is visible from the bottom membrane layer with flat fiber in Figure 9d, where there are no dirt particles. It can be observed that electrospun membranes have their top layer coated with more clay particles than the bottom layer. From this study, the efficiency of electrospun fibers can be proved by stopping all of the soil particles during filtration. This is similar to the results of Gopal et.al. [32], who studied the electrospun polyvinylidene fluride (PVDF) nanofibrous membranes for the fine filtration of polystyrene (PS) particles with different sizes of 1, 5, and 10 µm. The membrane surface of PVDF is illustrated before and after separation. Figure 7 shows that dust particles collected on the surface of the membrane of blend 3. Large particles of clay were deposited on the pores of the fibers, leading to closed fiber pores. This shows that soil and clays of different sizes were larger than the pore sizes in the membrane mat, remained primarily on the surface of the membrane mat, and also showed before treatment that there was unclear water and clear water after treatment. Figure 7 showed the morphology of PLLA nanofibers before and after filtration. For the blend 3 membrane before filtration, we could estimate that the diameter size of the nanofibers were about 1 µm–500 nm, the nanofiber mat from blend 3 membrane had dimensions of 40 mm × 35 mm × 0.3 mm, and the pore size was very small. After filtration, the sample filter was covered with various dust particles, colors, and some trace organic pollutants. We also observed that the PLLA membrane was still strong enough, and the surfaces of the nanofibers were attached to particles.

The clay particles of waste, fine sand, and slight soil were held and attached by electrospun fibers. It can be concluded that these nanofibers were manufactured from the PLLA blends as biomembranes and tested in water filtration to encourage the industry to use these membranes rather than the fiber from petrochemical plastics in the future. SEM images showed that sludge and dust collected on the surface of the PLLA electrospun fiber filter directly after the filtration tests in Figure 8 and Figure 9a–f. This membrane was tested by using a pump and flask with wastewater samples. When examining the blend 3 sample of the filter after the filtration process, we studied the surface of the filter by SEM from the front and back and the cross-section of the membrane.

It is interesting to note that most of the sand and clays were attached with fibers. The results showed that when the wastewater was filtered through the membranes, clean water was achieved. This can be a low-cost filtration approach for wastewater treatment for use in the agriculture field. The PLLA nanofiber membrane was successfully manufactured by the electrospinning method, and the resulting membranes were very strong during filtration testing when using wastewater to determine the effective filtration of the PLLA membrane. Water pollution in developing countries affects the public health of humans and the environment. Therefore, it is essential to develop environmentally friendly biopolymer, sustainable, and low-cost membranes. We observed that all of the particles and sand may have collected on the surface; a small portion may have collected on the existing half layer, and a very small part of the particles were present on the surface of the back membrane, which was almost clean when compared to the front.

## 4. Conclusions

The main objective of this study was to manufacture nanofibrous PLLA membranes using the electrospinning technique after improving its physical properties.

The experimental outcomes were as follows:

1. The addition of PPC, PHB, and TEC led to an improvement in the elongation at break, therefore reducing the tensile strength of the films. The fine structure of many PPC particles combined in the PLLA matrix improved the mechanical properties with very large stretching deformation in the PLLA blends (285%) when compared with pure PLLA (6%). The additives led to changes in the *T*_g_, *T*_cc_, and *T*_m_; therefore, the chain mobility increased.
2. The biodegradability test of the PLLA blend film was investigated using SEM in wastewater. The degradation at the surface began after more than two months. Degraded surfaces were observed, and some pores with different sizes were found.
3. The obtained fibers had a uniform, smooth morphology with a diameter between 500 nm and 3 µm, very small pores, and a fiber structure without beads. The electrospun nanofiber membranes can filter the nanosize and microsize element suspensions in wastewater. Using this method, liquid waste from the industry or home can be disposed of in a sustainable and economical way. Therefore, the PLLA biomembrane is a new solution to confirm clean water and preserve a sustainable environment in the future.

## Figures and Tables

**Table 1:** The blends prepared. PLLA: polylactic acid; PPC: polypropylene carbonate; PHB: poly (butylene carbonate); TEC: triethyl citrate.

		PLLA	PPC	PHB	TEC
1	blend 1	55	20	10	15
2	blend 2	50	20	10	20
3	blend 3	45	20	10	25
4	blend 4	40	20	10	30

**Table 2:** Thermal properties of pure PLLA and its blends at different concentrations. F.H.: first heating, S.H.: second heating.

	Pure PLLA	Blend 1	Blend 2	Blend 3	Blend 4
*T*_g_ (°C)	61	27	27	21	7
*T*_cc_ (°C)	135	100	102	92	78
*T*_m_ (°C) from S.H.	168	160	160	158	153
*T*_m_ (°C) from F.H.	171	158.8	158.7	156.8	152.7
ΔH_m_ (J/g)	7.1	29	24	25	22
ΔH_cc_ (J/g)	-	14	11	7	9
Χ (%)	8	23	20	30	25
